# Vaccination control programs for multiple livestock host species: an age-stratified, seasonal transmission model for brucellosis control in endemic settings

**DOI:** 10.1186/s13071-016-1327-6

**Published:** 2016-01-30

**Authors:** Wendy Beauvais, Imadidden Musallam, Javier Guitian

**Affiliations:** Veterinary Epidemiology, Economics and Public Health Group, The Royal Veterinary College, Hawkshead Lane, North Mymms, Hatfield AL9 7TA UK; London Centre for Neglected Tropical Disease Research, London, UK

**Keywords:** Modelling, Transmission, Brucellosis, Zoonosis, Control, Vaccination, Seasonality

## Abstract

**Background:**

*Brucella melitensis* causes production losses in ruminants and febrile disease in humans in Africa, Central Asia, the Middle East and elsewhere. Although traditionally understood to affect primarily sheep and goats, it is also the predominant *Brucella* species that affects cows in some endemic areas. Despite this, no licensed vaccine is available specifically for use against *B. melitensis* in cows. The mainstay of most control programs is vaccination of sheep and goats with a live vaccine, Rev-1. The aim of this study was to investigate how critical vaccination of cows might be, in order to control *B. melitensis* on a mixed sheep-and-cattle farm.

**Methods:**

A dynamic, differential-equation, age-structured, seasonal model with births and deaths, was used to investigate whether vaccination of both sheep and cattle had an impact on time to elimination of brucellosis on an individual mixed species farm, when compared to vaccination of sheep only. The model was a Susceptible-Exposed-Infectious-Recovered-Susceptible (SEIRS) model with an additional compartment for Persistently Infected (PI) individuals. Transmission parameters were fit based on a nation-wide probabilistic seroprevalence survey in Jordan.

**Results:**

The model predicted that it would take 3.5 years to eliminate brucellosis (to less than 0.5 % of adult sheep seropositive as a result of infection) on a mixed-species *B. melitensis*-endemic farm with the median field-study seroprevalence, following vaccination of both sheep and cattle, assuming a vaccine effectiveness of 80 %. Limiting the vaccination to sheep only, increased the time to 16.8 years. Sensitivity analysis showed that the finding that vaccination of cattle was of significant importance, was robust. Vaccine effectiveness had a strong influence on time to elimination.

**Conclusions:**

In the absence of further data, vaccination of cattle should be considered essential in *Brucella*-endemic settings where mixed small ruminant and cattle flocks predominate. Further evidence that *Brucella melitensis* predominates in cattle in Jordan, as opposed to *Brucella abortus,* is needed in order to validate this model. The results may be applicable to other mixed-species settings with similar livestock management practices. These methods may be applied to other pathogens affecting multiple livestock species or with seasonal transmission.

## Background

Brucellosis, a bacterial zoonosis, is the cause of febrile disease in humans and livestock production losses in many countries worldwide. Control has proved elusive in many areas, particularly where *Brucella melitensis*, the more pathogenic species for humans and small ruminants (sheep and goats), predominates, such as the Middle East [1, 2].

*Brucella spp.* are predominantly transmitted via direct contact with abortion and birth fluids of infected animals, and via consumption of unpasteurised milk or dairy products. Infection via the oral route, or via contact with conjunctiva or cuts in the skin is possible. In livestock, offspring of infected mothers can become persistently infected and remain seronegative until abortion occurs, when they are reported to seroconvert. Transmission via semen is also possible [3, 4].

Survival of *Brucella spp.* in the environment depends critically on humidity, temperature and exposure to UV light. Survival in ideal environments is reported to last up to 135 days, although a field study in spring in Montana, USA found that *Brucella abortus* survived in the environment for only 21-81 days, depending on the environment [5].

Human infection is almost invariably associated with an animal (mostly ruminant) source. In highly endemic areas, vaccination of the ruminant reservoir is the mainstay of brucellosis control programs, as well as general biosecurity and hygiene practices. However, in many countries where vaccination has been practised for many years, a high incidence in humans and livestock persists [6, 7]. This is a result of poor compliance due to legitimate concerns over safety of the live vaccines available, for humans and livestock (which commonly abort if pregnant); the limited efficacy of the vaccine; the need for careful storage and handling of the live vaccine for safety and to preserve its efficacy, as well as cost and availability of vaccine, particularly for smallholders who have the additional burden of low biosecurity farming systems and often poor access to medical and veterinary care.

Although *B. melitensis* has been traditionally thought of as a pathogen adapted to sheep and goats, and *B. abortus* adapted to cattle, cows are known to be susceptible to *B. melitensis*. In the Middle East and Central Asia, for example, high seroprevalence estimates are reported in cows and *B. melitensis* has been frequently isolated from cows [8–10]. It is common for cattle and small ruminants to co-graze or share pasture areas, and to be housed in the same building at night, in these regions.

Despite this situation, there has been no vaccine licensed for *B. melitensis* in cattle, and neither the safety nor efficacy of the small ruminant vaccine (Rev-1) has been thoroughly evaluated, neither has the *B. abortus* cattle vaccine for use against *B. melitensis*. This has been a stumbling block for policy-makers. In Jordan, for example, the official brucellosis program involves vaccination of small ruminants with Rev-1 vaccine, but no vaccination for cattle at all. However in practice, very little vaccination is practised at all [8]. There is no clear evidence for recommendations on vaccination of cattle in mixed-species settings endemic for *B. melitensis*.

Therefore, the aims of this study were firstly to simulate *Brucella melitensis* transmission on a single mixed sheep-and-cattle farm, incorporating: (a) heterogeneity in transmission parameters according to livestock species, age and season; and (b) seasonality in births of lambs. And, secondly, to use the model to compare vaccination of sheep-only with vaccination of sheep-and-cattle, in terms of time to elimination of *B. melitensis*.

Previous transmission models for brucellosis have included compartmental models with Susceptible-Infected (SI) [11] or Susceptible-Infected-Recovered (SIR) structures [12, 13]. However, they have not explicitly quantified levels of transmission between different ruminant species. Furthermore, transmission, which is highly dependent on abortion/birth events, having periodicity and seasonality, has not been linked in the model to the reproductive cycle of livestock. The age-structure of the herd has also not been taken into account; young animals are generally ignored in the model as they cannot become infectious. However they can become persistently infected, or infected while still juvenile, leading to infectiousness in adulthood. In this study we explore these three issues in a revised model, parameterized with field data from a nationwide seroprevalence study in Jordan, in which data on herd structures and reproductive parameters was also collected.

## Methods

### Seroprevalence survey

Data from a previously published nationwide seroprevalence study of randomly-sampled flocks and herds in Jordan was used to estimate median within-farm seroprevalence on *Brucella-*positive farms in Jordan [8]. It was assumed that the impact of *B. abortus* and *B. ovis* on median seroprevalence was negligible, and that *B. melitensis* was the cause of all seropositives, after adjusting for the sensitivity and specificity of the tests used. It was also assumed that the farm(s) with the median seroprevalence value exhibited endemic stability.

From the original dataset, all sheep-only farms (*n* = 203), cattle-only (*n* = 171) and mixed sheep-cattle farms (*n* = 27) were selected. On each farm, seven to nine female animals of each species had been milk-sampled (cows) or blood-sampled (sheep). The estimated true seroprevalence values at flock and herd levels were 22.2 % (95 % CI: 16.5–28.8) (sheep flocks), 18.1 % (95 % CI: 11–25.3) (cattle-only herds), and 38.5 % (95 % CI: 24.3–51.8) (mixed herds of cattle and small ruminants).

The tests used, and estimated sensitivity and specificity, are shown in Table 1. The number of female animals and the number of pregnancies in the previous year, by species were also recorded for each farm.

**Table 1:** Assumed sensitivity and specificity of tests used in the nationwide seroprevalence study of brucellosis in ruminants in Jordan [14]

Species	Material tested	Tests used	Sensitivity (combined)	Specificity (combined)
Cattle	Milk	indirect ELISA (Brucelisa; APHA Scientific)	0.988	0.9855
Sheep	Blood	Rose Bengal Test plus confirmatory testing using a competitive ELISA (Compelisa; APHA Scientific).	0.866725	0.999988

### Statistical analyses

The median within-farm seroprevalence was estimated separately for sheep-only, cattle-only and mixed sheep-cattle farms. Only female animals were included.

To account for uncertainty in the true within-farm seroprevalence resulting from both (1) imperfect tests and (2) sampling a variable fraction of animals on each farm in the study, a previously-developed Bayesian model (Beauvais et al. 2016, manuscript under review) was adapted to produce an uncertainty distribution of true within-herd seroprevalence on each farm. These distributions for each farm were repeatedly sampled, and the median seroprevalence amongst seropositive farms was calculated for each iteration, to produce an uncertainty distribution for the true median within-herd seroprevalence on seropositive farms in the study. The model was run for 1000 iterations (for sheep-only farms, cattle-only farms and mixed sheep-cattle farms). The sensitivity and specificity values were taken from a published meta-analysis [14]. There were multiple different ELISAs included in the meta-analysis, which were not identified by product name, and the mean of these values was used, after excluding an ELISA with exceptionally poor performance. The performance of the tests used in sheep and cattle are shown in Table 1.

To account for possible correlation between within-herd seroprevalence and two other parameters in the model: (1) the number of pregnancies per animal per year; and (2) the ratio of cows: sheep on mixed farms, these parameter values were identified for the farm with the median seroprevalence in each iteration of the model, to produce an uncertainty distribution for the true value of each parameter on the median-seroprevalence farm(s).

### Dynamic Brucellosis models

**Fig. 1:**
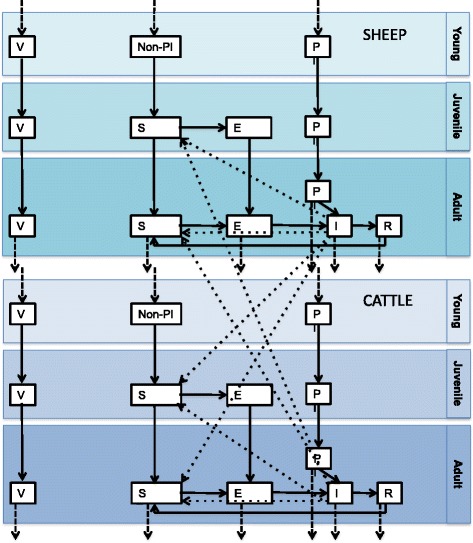
Diagram showing compartments in the transmission model (boxes) and transitions between compartments (solid arrows). Dashed arrows show births and deaths. PI = persistently infected, Non-PI = not persistently infected, V = vaccinated, S = susceptible, E = exposed (pre-infectious), I = Infectious and R = recovered. Dotted lines animals in one compartment infecting animals in another compartment. See section “Dynamic Brucellosis models” for more details

A dynamic, differential-equation, age-structured, seasonal model with births and deaths, was used to investigate whether vaccination of both sheep and cattle had an impact on time to elimination of brucellosis on an individual mixed-species farm, when compared to vaccination of sheep only. The model was a Susceptible-Exposed-Infectious-Recovered-Susceptible (SEIRS) model with an additional compartment for Persistently Infected (PI) individuals (Fig. 1). The model was run in R [15].

Only females are included in the model. The age categories are: young (0-9 months), when the animals cannot become infected; juvenile (9-12 months), when the animals can become infected but not infectious (Exposed or pre-infectious); and adult (> 12 months), when the animals can have a late abortion or give birth, at which time they can become Infectious (I). Aging and death occurs at a constant rate. Only adult animals die.

Replacement animals are assumed to be sourced *only* from amongst the youngstock born on the farm, at an annual rate equal to the death rate (m). Newborns that are sold off the farm are assumed to be sold before the age of 12 months, and so cannot become Infectious whilst on the farm. These animals are therefore not included in the model.

The population size remains constant from year to year, and replacement newborns enter the herd at a constant rate, however sheep births are limited to only 6 months of the year, the lambing season. The sheep replacement rate was therefore adjusted so that the mean annual replacement rate = annual death rate. Animals can be born either Susceptible (S), or Persistently Infected (PI) (1 % of newborns born to adults in the Exposed compartment). It is assumed that the birth rate is the same amongst all adult females (except in the Infectious compartment, in which case they have aborted/given birth in the last 4 months and cannot therefore abort/give birth). The proportion of newborn replacement animals that are PIs is therefore equal to 0.01 multiplied by the proportion of adult females that are in the Exposed compartment (excluding Infectious animals). PIs are seronegative but seroconvert when they have a late abortion/give birth, at which point they also become Infectious (I).

Susceptible (S) animals, once they enter the Juvenile age-group, can become Exposed (E) at a rate (r) proportional to the fraction of animals on the farm that are infectious (frequency-dependent transmission). Separate values of beta (the transmission coefficient) are used for sheep-to-sheep, cow-to-cow, sheep-to-cow and cow-to-sheep transmission. Homogenous mixing amongst different species and different age-groups is assumed, because close contact between animals is not necessary for transmission, rather it is assumed that the majority of transmission occurs through contact with placental fluid which may remain infectious for up to 4 months on pasture or bedding, which is shared between species and age-groups [5].

r for a given age-group (x), season (s) and species (i) is therefore given by:

$$ \left(\mathrm{bet}{\mathrm{a}}_{\mathrm{i},\mathrm{i}}*{{\mathrm{I}}^{\mathrm{a}}}_{\mathrm{i},\mathrm{s}}\kern0.5em  + \mathrm{bet}{\mathrm{a}}_{\mathrm{i},\mathrm{j}}*{{\mathrm{I}}^{\mathrm{a}}}_{\mathrm{j},\mathrm{s}}\right)*{{\mathrm{S}}^{\mathrm{x}}}_{\mathrm{i},\mathrm{s}}/\mathrm{N} $$

where:

beta_i,i_ represents the within-species transmission coefficient.

beta_i,j_ represents the between-species transmission coefficient.

I^a^_i,s_ represents the number of infectious adults of species i in season s.

I^a^_j,s_ represents the number of infectious adults of species j in season s.

S^x^_i,s_ represents the number of susceptible animals of a given age-group of a given species in a given season.

N represents the total number of animals of all ages and species on the farm.

Once in the Exposed (E) compartment, animals move into the Infectious (I) compartment at a rate v, the rate at which they have a late abortion/ give birth, which was assumed to equal the pregnancy rate reported by the farmers. (It was assumed that pregnancies resulting in early abortions, which are assumed not to be infectious as they result in reabsorption, are unlikely to have been detected by the farmer.)

Sheep can only have a late abortion/give birth during the 3 months preceding the lambing season, and the 6 months of the lambing season, and can therefore only move from the Exposed (E) to Infectious (I) compartments during these periods.

Animals move from the Infectious (I) to Recovered (R) compartment at a rate g, and remain seropositive until they re-join the Susceptible compartment at a rate z, when they become seronegative.

Mass vaccination occurs at a single time point, once endemic stability has been reached. A proportion (VE) of animals from each compartment, except the Infectious (I) and Persistently Infected (PI) compartments move into the Vaccinated compartments, where they remain until they die. (It is assumed that vaccination of Infectious and PI animals is ineffective.) Following this time-point, a proportion (VE) of newborn replacement animals enter directly into the Vaccinated (V) compartment, instead of the Susceptible (S) compartment. (It is assumed that replacement animals are vaccinated at some point between 0 and 9 months of age.)

The model is described by a set of differential equations shown in the Appendix. The parameters and values are described in Table 2.

**Table 2:** Parameters used in the SEIRS + PI transmission model for *B. melitensis*

Parameter (symbol)	Equation/value	Source
Age when susceptible to infection (a1)	9 months	[3]
Age when infectious abortion (due to Brucellosis) possible (a2)	12 months	Estimated from earliest conception date according to production data and earliest stage of pregnancy during which abortions due to brucellosis occur [3, 17, 18].
Age of death (a3)	Sheep: 39 months	Mean values of results of a survey of fifteen farmers in Jordan by co-author I. Musallam.
	Cattle: 51.36 months	
Duration of infectiousness (d1)	4 months	Estimated from period that bacteria are shed plus maximum survival of bacteria in the environment [5, 19].
Duration of protective immunity (d2)	8 months	Variability across reports and variability between animals [4].
Death and birth rate (m)	12/a3 (birth rate in sheep was adjusted for seasonality)	Calculated
Annual rate at which animals mature from “young” to “juvenile” (m1)	12/a1	Calculated
Annual rate at which animals mature from “juvenile” to “adult” (m2)	12/a2	Calculated
Annual rate of loss of infectiousness (v)	12/d1	calculated
Annual rate of loss of immunity (g)	12/d2	calculated
Vaccine effectiveness (VE)	Before intervention: 0. After intervention: 0.6, 0.8 and 0.9	Vaccine efficacy in experimental conditions: 80 % in ewes infected during first pregnancy and 62 % in ewes infected during second pregnancy.
Probability that a newborn is persistently infected given that the mother is seropositive (p)	0.01	[3]
Annual rate of abortion/giving birth (v)	Cows: 0.26	Estimated from pregnancies per animal per year, recorded during seroprevalence study (statistical analysis described below)
	Sheep: 0.33	
Rate at which animals recover from infection (g)	12/d1	calculated
Rate at which animals lose protective immunity (z)	12/d2	Calculated
Ratio of cows: sheep on a typical seropositive farm	2.73	Estimated from data recorded during seroprevalence study (statistical analysis described below)

### Fitting of transmission parameters

Transmission parameters were fit in three steps:

1. The model was set assuming there were zero cows on the farm, and fit to the most likely median within-farm seroprevalence estimated from the sheep-only farms in the seroprevalence study, assuming endemic stability. The best-fit transmission parameter was found by the least sum of squares (LSS) method (the squared difference between the endemic seroprevalence predicted by the model and the seroprevalence study), using the Brent method implemented in the Optim function in R. In this way, the sheep-to-sheep transmission parameter was obtained.
2. Step 1 was repeated for cattle-only farms, to obtain the cattle-to-cattle transmission parameter.
3. The model was set using the cattle: sheep ratio obtained from the seroprevalence study and the sheep-to-sheep and cattle-to-cattle transmission parameters. Cattle-to-sheep and sheep-to-cattle transmission parameters were obtained simultaneously using equal-weighted LSS, by the Nelder-Mead method implemented in the Optim function in R.

### Model output

The model was run using the fitted transmission parameters, once with vaccination of sheep and cattle, and once with vaccination of sheep only. In each case, we recorded the time to elimination of brucellosis on the farm, defined as reducing the proportion of seropositives (due to infection rather than vaccination) to <0.5 %. The threshold was chosen as a reasonable target for a brucellosis vaccination program on an individual farm of more than 200 animals. (On a smaller farm, a proportion of less than 0.5 % seropositives would imply eradication).

### Sensitivity analysis

The transmission parameter fitting process and model outputs were repeated for a range of values for parameters that we considered to be the most uncertain: duration of infectious period, duration of immune period, ratio of cows: sheep on the farm and vaccination effectiveness. Parameter values were selected based on biological plausibility or results of the field study (cows: sheep ratio). The results were plotted.

In order to investigate the impact of model structure assumptions on the conclusions, the transmission parameter fitting process and model outputs were also repeated using a simple Susceptible-Infected compartmental structure with no account for age or season.

### Scenario analysis

In addition, the fitted model was used to investigate how a change in the ratio of cows : sheep on the farm would affect the time to elimination, and the impact of cattle vaccination.

## Results

### Parameters estimated from seroprevalence study

The uncertainty distributions for median within-farm seroprevalence and associated cows: sheep ratio and pregnancy rates in the seroprevalence study are described in Table 3.

**Table 3:** Parameters estimated from the nationwide seroprevalence study of brucellosis in ruminants in Jordan

Farm type	Median within-farm seroprevalence on seropositive farms (5^th^, 25^th^, 75^th^ and 95^th^ percentiles of the uncertainty distribution)	Cow: sheep ratio on median-seroprevalence farm(s) (5^th^, 25^th^, 75^th^ and 95^th^ percentiles of the uncertainty distribution)	Annual pregnancy rates on median-seroprevalence farm(s) (5^th^, 25^th^, 75^th^ and 95^th^ percentiles of the uncertainty distribution)
Sheep-only	0.16 (0.15, 0.15, 0.19, 0.29)	-	0.35 (0, 0.35, 0.36, 0.36)
Cattle-only	0.22 (0.20, 0.21, 0.22, 0.32)	-	0.26 (0.25, 0.25, 0.27, 0.28)
Mixed sheep-cattle	Overall: 0.11 (0.06, 0.10, 0.14, 0.20)	2.53 (0.39, 2.00, 2.73, 3.56)	Sheep: 0.33 (0.32, 0.33, 0.35, 0.36)
	Sheep: 0.13 (0, 0.11, 0.17, 0.47)		Cattle: 0.26 (0.25, 0.26, 0.27, 0.31)
	Cattle: 0.098 (0, 0.09, 0.11, 0.23)		

### Model fit

Good-fit transmission parameters were obtained for all models, with model sum of squares ranging from 1.26x10^−17^ to 1.48 x10^−15^ for sheep-only models; 1.43 x10^−20^ to 1.56 x10^−17^ for cow-only models; and 3.49 x10^−13^ to 1.42 x10^−10^ for mixed sheep-cattle models.

### Model predictions

**Fig. 2:**
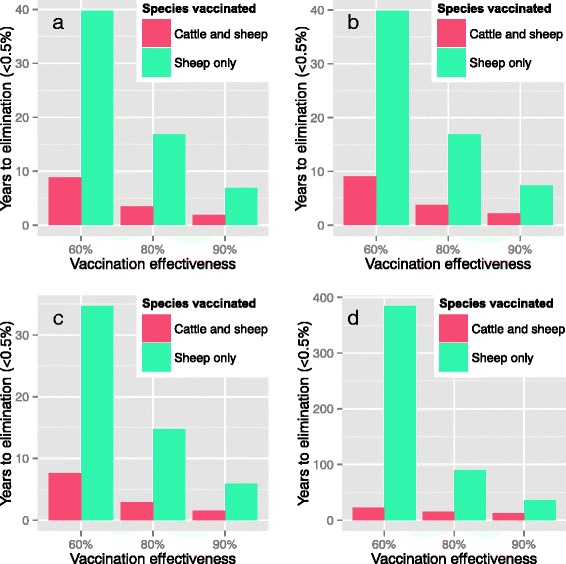
Time to elimination of sheep brucellosis (to <0.5 % seropositive due to infection as opposed to vaccination) after mass vaccination followed by vaccination of replacements on a mixed sheep-cattle farm. The transmission model was fit to median seroprevalence on endemic farms in a randomly sampled survey in Jordan, using the SEIR+PI structure, seasonality in lambing period and sheep-sheep and sheep-cow transmission, and an age-structure. a. A mean immune (Recovered) period of 8 months, and an infectious period of 4 months was assumed. b. A mean immune (Recovered) period of 20 months, and an infectious period of 4 months was assumed. c. A mean immune (Recovered) period of 11.5 months, and an infectious period of 0.5 months was assumed. d. The transmission model was fit to median seroprevalence on endemic farms in a randomly sampled survey in Jordan, using the SI structure, no seasonality or agestructure

On a single mixed-species farm, assuming an infectious period of 4 months, a recovery period of 8 months, and a vaccine effectiveness of 80 %, sheep-only vaccination resulted in elimination of sheep brucellosis (to <0.5 % of adults seropositive due to infection as opposed to vaccination) in 16.8 years, whereas it took only 3.5 years with vaccination of both sheep and cattle (Fig. 2a).

### Sensitivity analysis

Assuming a recovery period of 20 months or an infectious period of 0.5 months and a recovery period of 11.5 months had a relatively small impact on the results (Fig. 2b and c). Vaccine effectiveness had an impact on the overall times to elimination, but the difference between sheep-only and sheep-and-cattle vaccination remained large.

A simple SI model with no seasonality or age-structure was fit to the same data. The time to elimination increased greatly, however the finding that vaccination of cattle would be necessary to eliminate infection in sheep appeared to be robust (Fig. 2d).

**Fig. 3:**
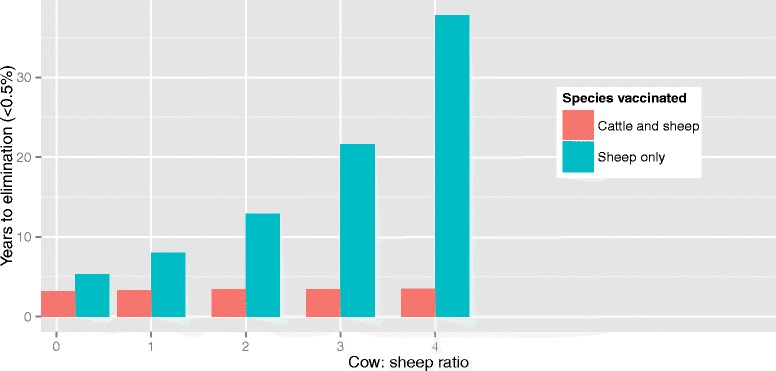
Time to elimination of sheep brucellosis (to <0.5 % seropositive due to infection as opposed to vaccination) after mass vaccination followed by vaccination of replacements on a mixed sheep-cattle farm assuming various ratios of cows: sheep on the farm

Assuming different ratios of cows : sheep on the farm had a minor impact on the time to elimination with vaccination of both cattle and sheep (Fig. 3). Vaccination of sheep only resulted in an increase of 2.1 years to elimination, even when the ratio of cows: sheep on the farm was 0.2, and the difference increased with an increasing ratio of cows: sheep.

### Scenario analysis

**Fig. 4:**
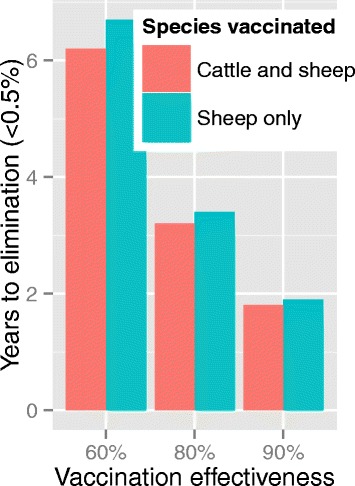
Time to elimination of sheep brucellosis (to <0.5 % seropositive due to infection as opposed to vaccination) after mass vaccination followed by vaccination of replacements on a mixed sheep-cattle farm, using the fitted transmission model, assuming the ratio of cows: sheep was changed to 1:10

The fitted model was used to investigate potential changes to the production system that may affect the transmission dynamics. When the ratio of cows to sheep was reduced to 1:10, the time to elimination decreased, and the effect of vaccinating cattle was minor (Fig. 4). When the lambing season was reduced from 6 months to 1 month, transmission ceased altogether after a single epidemic peak.

## Discussion

The model predicted that it would take several years to eliminate brucellosis on a typical mixed-species *B. melitensis*-endemic farm following vaccination, even for the most optimistic scenarios. Limiting the vaccination to sheep-only increased the time to elimination greatly, such that the economic payback for vaccination may be so delayed as to make the program impractical and unjustifiable.

Elimination was defined as reducing the number of seropositives due to infection to <0.5 %, as it was considered a meaningful target for a brucellosis vaccination program on an individual farm. For the final stages of eradication programs, test and slaughter is often used. One reason for this is that in practice there is currently no good way of monitoring the effectiveness of *Brucella* Rev-1 or S19 vaccination programs – apart from ensuring a high percentage are seropositive following vaccination. Seropositives as a result of infection cannot yet be reliably distinguished from animals vaccinated with Rev-1 or S19 vaccines [3].

According to the model assumptions, new infections would cease several months to a year before the final seropositive either died or became seronegative. Stochastic fade-out could prove to be an important factor in time to elimination of brucellosis, particularly on small farms. This could be investigated further using a stochastic model. Further, on farms of fewer than 200 animals, transmission would cease sooner than predicted, because a threshold of <0.5 % seropositives were used. Nevertheless, the key finding- that vaccination of cattle in addition to sheep can be expected to have a significant impact- would still hold on smaller farms.

There are some important assumptions that could have lead to an over- or under-estimation of the transmission parameter. It was assumed that the median seroprevalence farms exhibited endemic stability, however if they were in fact at the beginning of an epidemic, we could have over-estimated transmission (or potentially under-estimated transmission if at the end of an epidemic). However, *B. melitensis* has been endemic in Jordan decades and in the seroprevalence study seropositives were found even on farms that reported purchasing no new animals in the preceding year. There is commonly limited contact between farms in Jordan (particularly cattle-only and mixed farms; sheep-only farms to a lesser extent). These factors lead to a higher likelihood that there was a state of endemic stability on the median-seroprevalence farm(s).

There is uncertainty, and probably variation, in the true infectious period and immune period for brucellosis, however when a variety of different assumptions about these parameters were made, there was a limited impact on the overall conclusions. Furthermore, considering that infectiousness is primarily related to abortion/birth events, the time from one infectious period to the next in a single animal is limited by the reproductive cycle, making the exact immune period less important in the model. In addition, the life span of the animals is relatively short, making the length of the immune period even less important.

In order to investigate the importance of the assumptions about the model structure, the work was repeated using a simple Susceptible-Infected (SI) structure, ignoring seasonal and age dependence. This resulted in very long predicted times to elimination, but the finding that vaccination of cattle was important was found to be robust.

When simulating the effectiveness of the vaccination program, it was assumed that there was no re-introduction of infection via contact with other herds or introductions of new animals. This may be an important factor in considering applying these results to a national program. The results should be interpreted as a “best-case scenario” for a single farm. Eliminating brucellosis from a region can be expected to be an even more lengthy process.

Accepting these limitations to the model, the findings suggest that the role of cattle in transmission of *B. melitensis* in mixed-species endemic settings cannot be ignored, and it is likely that vaccination of small ruminants alone may be futile in many cases, in terms of eliminating the infection. A caveat is that vaccination of small ruminants may significantly reduce infectiousness to humans, and vaccination could be justifiable as a public health measure, even if it is not effective in eliminating infection from farms [16]. However, vaccinating sheep and cows is likely to be a much more effective public health measure, in many cases.

Vaccine effectiveness was found to be critical to time to elimination. Vaccine effectiveness is a result of both vaccine efficacy and the thoroughness of the vaccination program as well as the degree to which the live vaccine is stored and handled properly to preserve its efficacy. The Rev-1 vaccine has been shown to have an efficacy of approximately 80 % in eliminating infectiousness, in experimental conditions. However the efficacy decreased in subsequent pregnancies. In practice the vaccine effectiveness may therefore be much lower, although vaccine immunity could in theory be boosted by exposure to natural infection or the Rev-1 vaccine given to replacement animals in subsequent years, which can be shed. The scenarios modelled using a vaccine effectiveness of 90 % are therefore highly optimistic, particularly if applied to a regional vaccination program, which entails more logistical difficulties. The vaccine effectiveness values applied to cattle are theoretical values, as there was no validation data available on vaccines against *B. melitensis* in cattle.

The study suggests that the length of the lambing season may have an important impact on the dynamics of *B. melitensis.* Lambing seasons vary in length according to geographical location and breed, and can also be deliberately managed to produce a shorter or longer lambing season. This could have implications for the transmission of several infectious diseases of small ruminants. Transmission models of brucellosis and other diseases for which transmission is related to reproductive status should take this into consideration.

## Conclusions

In conclusion, until now a relatively simplistic approach to brucellosis control has been taken, based on the underlying assumption that *B. abortus* infects cattle and *B. melitensis* infects small ruminants, and largely ignoring transmission between species. Although this simplification is probably justified in many settings, it may be inappropriate where mixed-species herds are common. In the absence of further data, vaccination of cattle should be considered as potentially essential for control of *B. melitensis* in settings where mixed small ruminant and cattle flocks exist, particularly where the ratio of cows to sheep is high. Maximising vaccine coverage and vaccine efficacy is critical to the success of *B. melitensis* control programs. Given the long predicted time to elimination with vaccination alone, other biosecurity practices such as disinfection of calving and lambing areas may have a critical impact on the success of control. Further evidence that *Brucella melitensis* predominates in cattle in Jordan, as opposed to *Brucella abortus,* is needed in order to validate these results. The results may be applicable to other mixed-species settings with similar livestock management practices.

### Ethical approval

Ethical approval for the seroprevalence study was granted by the Ethics and Welfare Committee of the Royal Veterinary College.

## Appendix

The differential equations used for the model are as follows:

Young animals:

$$ \begin{array}{l}\mathrm{d}{{\mathrm{S}}^{\mathrm{y}}}_{\mathrm{i},\mathrm{s}}/\mathrm{d}\mathrm{t} = \left(1\hbox{-} \mathrm{V}\mathrm{E}\right)*{\mathrm{m}}_{\mathrm{i},\mathrm{s}}*\left[1\hbox{-} \left(\mathrm{p}*{{\mathrm{E}}^{\mathrm{a}}}_{\mathrm{i},\mathrm{s}}/\ \left({{\mathrm{S}}^{\mathrm{a}}}_{\mathrm{i},\mathrm{s}} + {{\mathrm{E}}^{\mathrm{a}}}_{\mathrm{i},\mathrm{s}} + \mathrm{P}{{\mathrm{I}}^{\mathrm{a}}}_{\mathrm{i},\mathrm{s}} + {{\mathrm{V}}^{\mathrm{a}}}_{\mathrm{i},\mathrm{s}}\right)\right)\right]\ \hbox{--}\ \mathrm{m}{1}_{\mathrm{i},\mathrm{s}}*{{\mathrm{S}}^{\mathrm{y}}}_{\mathrm{i},\mathrm{s}}\\ {}\mathrm{d}\mathrm{P}{{\mathrm{I}}^{\mathrm{y}}}_{\mathrm{i},\mathrm{s}}/\mathrm{d}\mathrm{t} = {\mathrm{m}}_{\mathrm{i},\mathrm{s}}*\left(\mathrm{p}*{{\mathrm{E}}^{\mathrm{a}}}_{\mathrm{i},\mathrm{s}}/\ \left({{\mathrm{S}}^{\mathrm{a}}}_{\mathrm{i},\mathrm{s}} + {{\mathrm{E}}^{\mathrm{a}}}_{\mathrm{i},\mathrm{s}} + \mathrm{P}{{\mathrm{I}}^{\mathrm{a}}}_{\mathrm{i},\mathrm{s}} + {{\mathrm{V}}^{\mathrm{a}}}_{\mathrm{i},\mathrm{s}}\right)\right)\ \hbox{--}\ \mathrm{m}{1}_{\mathrm{i},\mathrm{s}}*\mathrm{P}{{\mathrm{I}}^{\mathrm{y}}}_{\mathrm{i},\mathrm{s}}\\ {}\mathrm{d}{{\mathrm{V}}^{\mathrm{y}}}_{\mathrm{i},\mathrm{s}}/\mathrm{d}\mathrm{t} = \mathrm{V}\mathrm{E}*{\mathrm{m}}_{\mathrm{i},\mathrm{s}}*\left[1\hbox{-} \left(\mathrm{p}*{{\mathrm{E}}^{\mathrm{a}}}_{\mathrm{i},\mathrm{s}}/\ \left({{\mathrm{S}}^{\mathrm{a}}}_{\mathrm{i},\mathrm{s}} + {{\mathrm{E}}^{\mathrm{a}}}_{\mathrm{i},\mathrm{s}} + \mathrm{P}{{\mathrm{I}}^{\mathrm{a}}}_{\mathrm{i},\mathrm{s}} + {{\mathrm{V}}^{\mathrm{a}}}_{\mathrm{i},\mathrm{s}}\right)\right)\right]\ \hbox{--}\ \mathrm{m}{1}_{\mathrm{i},\mathrm{s}}*{{\mathrm{V}}^{\mathrm{y}}}_{\mathrm{i},\mathrm{s}}\end{array} $$

Juvenile animals:

$$ \begin{array}{l}\mathrm{d}{{\mathrm{S}}^{\mathrm{j}\mathrm{uv}}}_{\mathrm{i},\mathrm{s}}/\mathrm{d}\mathrm{t} = \mathrm{m}{1}_{\mathrm{i},\mathrm{s}}*{{\mathrm{S}}^{\mathrm{y}}}_{\mathrm{i},\mathrm{s}}\hbox{--}\ \mathrm{m}{2}_{\mathrm{i},\mathrm{s}}*{{\mathrm{S}}^{\mathrm{j}\mathrm{uv}}}_{\mathrm{i},\mathrm{s}}\hbox{--}\ \left(\mathrm{bet}{\mathrm{a}}_{\mathrm{i},,\mathrm{i}}*{{\mathrm{I}}^{\mathrm{a}}}_{\mathrm{i},\mathrm{s}} + \mathrm{bet}{\mathrm{a}}_{\mathrm{i},,\mathrm{j}}*{{\mathrm{I}}^{\mathrm{a}}}_{\mathrm{j},\mathrm{s}}\right)*{{\mathrm{S}}^{\mathrm{j}\mathrm{uv}}}_{\mathrm{i},\mathrm{s}}/\mathrm{N}\\ {}\mathrm{d}{{\mathrm{E}}^{\mathrm{j}\mathrm{uv}}}_{\mathrm{i},\mathrm{s}}/\mathrm{d}\mathrm{t} = \left(\mathrm{bet}{\mathrm{a}}_{\mathrm{i},,\mathrm{i}}*{{\mathrm{I}}^{\mathrm{a}}}_{\mathrm{i},\mathrm{s}} + \mathrm{bet}{\mathrm{a}}_{\mathrm{i},,\mathrm{j}}*{{\mathrm{I}}^{\mathrm{a}}}_{\mathrm{j},\mathrm{s}}\right)*{{\mathrm{S}}^{\mathrm{j}\mathrm{uv}}}_{\mathrm{i},\mathrm{s}}/\mathrm{N}\ \hbox{--}\ \mathrm{m}{2}_{\mathrm{i},\mathrm{s}}*{{\mathrm{E}}^{\mathrm{j}\mathrm{uv}}}_{\mathrm{i},\mathrm{s}}\\ {}\mathrm{d}\mathrm{P}{{\mathrm{I}}^{\mathrm{j}\mathrm{uv}}}_{\mathrm{i},\mathrm{s}}/\mathrm{d}\mathrm{t} = \mathrm{m}{1}_{\mathrm{i},\mathrm{s}}*\mathrm{P}{{\mathrm{I}}^{\mathrm{y}}}_{\mathrm{i},\mathrm{s}}\hbox{--}\ \mathrm{m}{2}_{\mathrm{i},\mathrm{s}}*\mathrm{P}{\mathrm{I}}_{\mathrm{i},\mathrm{s}}^{\mathrm{j}\mathrm{uv}}\\ {}\mathrm{d}{{\mathrm{V}}^{\mathrm{j}\mathrm{uv}}}_{\mathrm{i},\mathrm{s}}/\mathrm{d}\mathrm{t} = \mathrm{m}{1}_{\mathrm{i},\mathrm{s}}*{{\mathrm{V}}^{\mathrm{y}}}_{\mathrm{i},\mathrm{s}}\hbox{--}\ \mathrm{m}{2}_{\mathrm{i},\mathrm{s}}*{{\mathrm{V}}^{\mathrm{j}\mathrm{uv}}}_{\mathrm{i},\mathrm{s}}\end{array} $$

Adult animals:

$$ \begin{array}{l}\mathrm{d}{{\mathrm{S}}^{\mathrm{a}}}_{\mathrm{i},\mathrm{s}}/\mathrm{d}\mathrm{t} = \mathrm{m}{2}_{\mathrm{i},\mathrm{s}}*{{\mathrm{S}}^{\mathrm{j}\mathrm{uv}}}_{\mathrm{i},\mathrm{s}}\hbox{--}\ {\mathrm{m}}_{\mathrm{i},\mathrm{s}}{{\mathrm{S}}^{\mathrm{a}}}_{\mathrm{i},\mathrm{s}}\hbox{--}\ \left(\mathrm{bet}{\mathrm{a}}_{\mathrm{i},\mathrm{i}}*{{\mathrm{I}}^{\mathrm{a}}}_{\mathrm{i},\mathrm{s}} + \mathrm{bet}{\mathrm{a}}_{\mathrm{i},\mathrm{j}}*{{\mathrm{I}}^{\mathrm{a}}}_{\mathrm{j},\mathrm{s}}\right)*{{\mathrm{S}}^{\mathrm{a}}}_{\mathrm{i},\mathrm{s}}/\mathrm{N} + \mathrm{z}*{{\mathrm{R}}^{\mathrm{a}}}_{\mathrm{i},\mathrm{s}}\\ {}\mathrm{d}{{\mathrm{E}}^{\mathrm{a}}}_{\mathrm{i},\mathrm{s}}/\mathrm{d}\mathrm{t} = \mathrm{m}{2}_{\mathrm{i},\mathrm{s}}*{{\mathrm{E}}^{\mathrm{j}\mathrm{uv}}}_{\mathrm{i},\mathrm{s}}\hbox{--}\ {\mathrm{m}}_{\mathrm{i}\mathrm{s}}{{\mathrm{E}}^{\mathrm{a}}}_{\mathrm{i},\mathrm{s}} + \left(\mathrm{bet}{\mathrm{a}}_{\mathrm{i},\mathrm{i}}*{{\mathrm{I}}^{\mathrm{a}}}_{\mathrm{i},\mathrm{s}} + \mathrm{bet}{\mathrm{a}}_{\mathrm{i},\mathrm{j}}*{{\mathrm{I}}^{\mathrm{a}}}_{\mathrm{j},\mathrm{s}}\right)*{{\mathrm{S}}^{\mathrm{a}}}_{\mathrm{i},\mathrm{s}}/\mathrm{N}\ \hbox{--}\ \mathrm{v}*{{\mathrm{E}}^{\mathrm{a}}}_{\mathrm{i},\mathrm{s}}\\ {}\mathrm{d}{{\mathrm{I}}^{\mathrm{a}}}_{\mathrm{i},\mathrm{s}}/\mathrm{d}\mathrm{t} = \mathrm{v}*{{\mathrm{E}}^{\mathrm{a}}}_{\mathrm{i},\mathrm{s}} + \mathrm{v}*\mathrm{P}{{\mathrm{I}}^{\mathrm{a}}}_{\mathrm{i},\mathrm{s}}\hbox{--}\ \mathrm{g}*{{\mathrm{I}}^{\mathrm{a}}}_{\mathrm{i},\mathrm{s}}\hbox{--}\ {\mathrm{m}}_{\mathrm{i},\mathrm{s}}{{\mathrm{I}}^{\mathrm{a}}}_{\mathrm{i},\mathrm{s}}\\ {}\mathrm{d}{{\mathrm{R}}^{\mathrm{a}}}_{\mathrm{i},\mathrm{s}}/\mathrm{d}\mathrm{t} = \mathrm{g}*{{\mathrm{I}}^{\mathrm{a}}}_{\mathrm{i},\mathrm{s}}\hbox{--}\ {\mathrm{m}}_{\mathrm{i}\mathrm{s}}{{\mathrm{R}}^{\mathrm{a}}}_{\mathrm{i},\mathrm{s}}\hbox{-}\ \mathrm{z}*{{\mathrm{R}}^{\mathrm{a}}}_{\mathrm{i},\mathrm{s}}\\ {}\mathrm{d}\mathrm{P}{{\mathrm{I}}^{\mathrm{a}}}_{\mathrm{i},\mathrm{s}}/\mathrm{d}\mathrm{t} = \mathrm{m}{2}_{\mathrm{i}\mathrm{s}}*\mathrm{P}{{\mathrm{I}}^{\mathrm{j}\mathrm{uv}}}_{\mathrm{i},\mathrm{s}}\hbox{--}\ {\mathrm{m}}_{\mathrm{i},\mathrm{s}}\mathrm{P}{{\mathrm{I}}^{\mathrm{a}}}_{\mathrm{i},\mathrm{s}}\hbox{-}\ \mathrm{v}*\mathrm{P}{{\mathrm{I}}^{\mathrm{a}}}_{\mathrm{i},\mathrm{s}}\\ {}\mathrm{d}{{\mathrm{V}}^{\mathrm{a}}}_{\mathrm{i},\mathrm{s}}/\mathrm{d}\mathrm{t} = \mathrm{m}{2}_{\mathrm{i}\mathrm{s}}*{{\mathrm{V}}^{\mathrm{j}\mathrm{uv}}}_{\mathrm{i},\mathrm{s}}\hbox{--}\ {\mathrm{m}}_{\mathrm{i},\mathrm{s}}*{{\mathrm{V}}^{\mathrm{a}}}_{\mathrm{i},\mathrm{s}}\end{array} $$

$$ \mathrm{N} = \Sigma \left({{\mathrm{S}}^{\mathrm{y}}}_{\mathrm{s}} + \mathrm{P}{{\mathrm{I}}^{\mathrm{y}}}_{\mathrm{s}} + {{\mathrm{V}}^{\mathrm{y}}}_{\mathrm{s}} + {{\mathrm{S}}^{\mathrm{j}}}_{\mathrm{s}} + {{\mathrm{E}}^{\mathrm{j}}}_{\mathrm{s}} + \mathrm{P}{{\mathrm{I}}^{\mathrm{j}}}_{\mathrm{s}} + {{\mathrm{V}}^{\mathrm{j}}}_{\mathrm{s}} + {{\mathrm{S}}^{\mathrm{a}}}_{\mathrm{s}} + {{\mathrm{E}}^{\mathrm{a}}}_{\mathrm{s}} + {{\mathrm{I}}^{\mathrm{a}}}_{\mathrm{s}} + {{\mathrm{R}}^{\mathrm{a}}}_{\mathrm{s}} + \mathrm{P}{{\mathrm{I}}^{\mathrm{a}}}_{\mathrm{s}} + {{\mathrm{V}}^{\mathrm{a}}}_{\mathrm{s}}\right) $$

where:

_s_ denotes season

_i_ denotes species

^y^ denotes young

^juv^ denotes juvenile

^a^ denotes adult

NI represents young animals that are not persistently infected

VE represents vaccination effectiveness

m represents the death and birth rate

p represents the probability that a newborn is persistently infected given that the mother is seropositive

E represents animals in the exposed (pre-infectious) compartment

S represents susceptibles

PI represents persistently infected animals

V represents vaccinated animals

m1 represents the annual rate at which animals mature from “young” to “juvenile”

m2 represents the annual rate at which animals mature from “juvenile” to “adult”

beta_i,i_ represents within-species transmission and beta_i,j_ represents between-species transmission coefficient.

I represents infectious animals

N represents the total population

z represents the rate at which animals lose protective immunity

R represents immune animals

v represents the annual rate of loss of infectiousness

g represents the annual rate of loss of immunity
